# Salivary cotinine and S100A8/A9 levels in children exposed to environmental tobacco smoke: a case-control study

**DOI:** 10.1590/1678-7765-2025-0840

**Published:** 2026-05-15

**Authors:** Arzu KOCKANAT, Sukran ACIPINAR

**Affiliations:** 1 Sivas Cumhuriyet University Faculty of Dentistry Department of Pedodontics Sivas Türkiye Sivas Cumhuriyet University, Faculty of Dentistry, Department of Pedodontics, Sivas, Türkiye.; 2 Sivas Cumhuriyet University Faculty of Dentistry Department of Periodontology Sivas Türkiye Sivas Cumhuriyet University, Faculty of Dentistry, Department of Periodontology, Sivas,Türkiye.

**Keywords:** Biomarkers, Children, Cotinine, Inflammation, Smoking

## Abstract

Exposure to environmental tobacco smoke (ETS) poses significant risks to children’s oral health by inducing oxidative stress and triggering inflammatory responses. Even in the absence of clinically evident oral inflammation, early biological changes can be detected in children exposed to ETS.

**Objectives:**

This study aimed to evaluate salivary cotinine and S100A8/A9 levels in children exposed to ETS and to examine the relationship between exposure intensity and inflammatory biomarker expression.

**Methodology:**

This observational case-control study included 150 systemically healthy children aged 6–12 years. ETS exposure was determined using parent-reported questionnaires. Clinical parameters, including plaque index, gingival index, and probing pocket depth, were recorded. Unstimulated saliva samples were analyzed for cotinine, S100A8, and S100A9 using ELISA kits. Group comparisons were conducted using the Mann–Whitney U test, and correlations were assessed using Spearman’s rank correlation coefficient. Statistical significance was set at p<0.05.

**Results:**

ETS-exposed and non-exposed groups did not differ significantly in age, sex, socioeconomic status, oral hygiene habits, or clinical periodontal parameters (p>0.05). However, salivary cotinine, S100A8, and S100A9 levels were significantly higher in ETS-exposed children (p<0.05). Cotinine demonstrated a moderate positive correlation with S100A8, while its correlation with S100A9 was weak and non-significant. Exposure intensity was positively associated with cotinine and S100A8 levels, but not with S100A9.

**Conclusion:**

ETS exposure in children is associated with early biochemical signs of inflammation, even in the absence of clinically detectable periodontal changes. Elevated salivary cotinine and S100A8 levels highlight the potential utility of salivary biomarkers for identifying subclinical effects of passive smoking. These findings underscore the importance of interventions aimed at reducing children’s exposure to ETS to protect long-term oral health. Clinical trial registration: NCT06791707

## Graphical abstract

**Figure:**
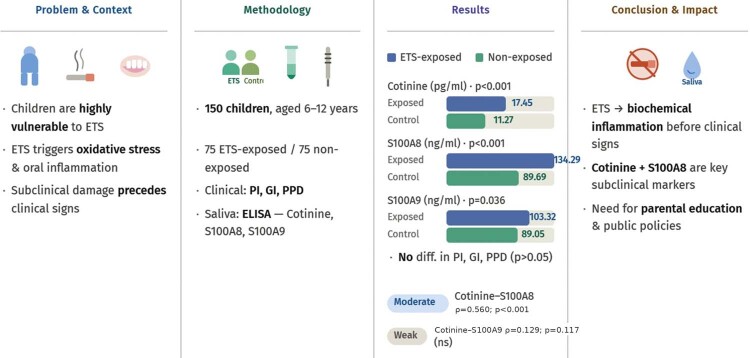

## Introduction

Exposure to tobacco smoke poses significant systemic and local health risks for both active smokers and individuals involuntarily exposed to environmental tobacco smoke (ETS).^1,2^ In addition to its well-known respiratory effects, ETS can also adversely affect oral tissues. Toxic compounds in tobacco smoke, such as nicotine, carbon monoxide, free radicals, and heavy metals, induce oxidative stress at the cellular level. Reactive oxygen species generated during this process can cause lipid peroxidation of cell membranes, damage proteins and DNA, and ultimately compromise tissue integrity. Elevated oxidative stress is recognized as a key trigger in the initiation and perpetuation of inflammatory responses.^3,4^ Moreover, ETS can impair antioxidant defense mechanisms, disrupt immune cell function, and induce epigenetic alterations, thereby further increasing the vulnerability of oral tissues.^5^

Children are particularly vulnerable to the harmful effects of ETS compared to adults. This increased susceptibility is due to several factors, including their developing immune systems, narrower airways, higher respiratory rates relative to body weight, and limited capacity to metabolize and eliminate toxic compounds.^6,7^ Furthermore, children living in households where smoking is prevalent are exposed to tobacco smoke for prolonged periods, leading to cumulative effects.^8^

Nicotine in tobacco smoke is rapidly absorbed by the lungs and extensively metabolized in the liver to its primary immediate metabolite, cotinine. Cotinine has a significantly longer elimination half-life than nicotine—typically 16–20 hours—making it a more stable and practical biomarker for assessing recent tobacco exposure. Because cotinine is specific to tobacco and nicotine exposure and is readily detectable in blood, urine, and saliva, it is widely used to confirm both active smoking and involuntary ETS exposure. In pediatric research, salivary cotinine measurement is widely used as a noninvasive and feasible alternative to blood sampling.^9,10^

Within the inflammatory process, the proteins S100A8 and S100A9 are essential. These calcium-binding proteins, primarily released by neutrophils and monocytes, act as alarmins and are highly expressed during the early stages of inflammation. The S100A8/A9 heterodimer (calprotectin) regulates immune cell migration, stimulates cytokine release, and amplifies oxidative stress, thereby sustaining the inflammatory cycle. Elevated levels of S100A8/A9 have been associated with both systemic and localized inflammatory diseases and are considered diagnostic biomarkers as well as potential therapeutic targets.^11,12^

Oral inflammatory responses result from a complex interaction between microbial dental plaque and the host immune system. Although clinical signs such as gingival bleeding, increased probing depth, and attachment loss characterize overt periodontal disease, the inflammatory process often begins at a subclinical stage, before any visible symptoms appear. Therefore, assessing whether ETS exposure influences salivary inflammatory biomarkers in children is essential for detecting early subclinical oral inflammation and identifying potential risk factors prior to the development of clinical disease.^13,14^ To the best of our knowledge, no previous study has examined the relationship between ETS exposure and salivary S100A8/A9 levels in children. This study aims to compare salivary cotinine and S100A8/A9 levels in children exposed to ETS with those of unexposed children, assessing early biochemical effects of tobacco smoke on oral inflammation.

## Methodology

### Study design and ethical approval

This research was conducted as an observational case-control study and reported in accordance with the Strengthening the Reporting of Observational Studies in Epidemiology (STROBE) guidelines.^15^ Ethical approval was obtained from the Erzincan Binali Yıldırım University Clinical Research Ethics Committee (decision number: 2024-11/03; date: August 1, 2024). Prior to participation, both the children and their parents were fully informed about the study objectives and procedures, and written informed consent was obtained from the parents or legal guardians. The study was prospectively registered on ClinicalTrials.gov (Identifier: NCT06791707) to ensure methodological transparency and compliance with ethical research standards.

### Sample size and study population

A power analysis was conducted using G Power 3.1 to determine the minimum sample size required to detect a significant difference between groups in salivary cotinine levels, which was the primary outcome. Based on pilot study data (Group 1: 10.66±3.25 pg/ml; Group 2: 13.72±5.23 pg/ml; n=10 per group), the calculated effect size (Cohen’s d) was 0.93, indicating a large effect. The analysis showed that at least 52 participants per group were needed to achieve 95% power at an alpha level of 0.05. Accordingly, 150 children (75 in each group) were included, exceeding the minimum required sample size to enhance representativeness and statistical reliability.

This study included children and their parents who presented to the Department of Pediatric Dentistry at Sivas Cumhuriyet University Faculty of Dentistry for routine dental care and consented to participate. Children aged 6–12 years who were systemically healthy and had a body mass index (BMI) within the normal range for their age and gender were included. Children with chronic systemic or genetic diseases, obesity or underweight status, a history of infection or medication use within the past month, or who had received periodontal treatment within the previous six months were excluded, as these conditions could potentially affect immune responses or inflammatory parameters. To minimize selection bias and ensure representativeness, participants were consecutively recruited from April 2025 to September 2025 until the target sample size was achieved.

### Data collection and group allocation

Parents completed a structured, interviewer-administered questionnaire that collected information on demographic characteristics (age, sex, parental education, occupation, and income), children’s oral hygiene practices (availability of a toothbrush and toothpaste, toothbrushing frequency, and routine dental visits), and household smoking habits (parental smoking status, duration of smoking, and presence of other smokers in the home). Additionally, daily smoking exposure intensity quantified based on parental self-report of the average number of cigarettes smoked per day (<10, 10–20, >20).

Based on the questionnaire responses, children with at least one parent who reported regular and consistent smoking inside the home were classified as the ETS-exposed group, while those whose parents were non-smokers and reported no household exposure to tobacco smoke were assigned to the control group.

### Periodontal clinical assessment

All children underwent a comprehensive intraoral examination conducted by a single calibrated periodontologist under standardized clinical conditions, using artificial light, a mouth mirror, and a WHO periodontal probe. To ensure intra-examiner reliability, a pilot calibration was performed on 20 children prior to data collection, with re-examinations conducted one week apart. The Cohen’s kappa coefficient (κ>0.80) indicated a high level of agreement. Additionally, the examiner was blinded to the questionnaire results and group allocation to minimize observer bias.

The gingival index (GI) was used to assess the severity of gingival inflammation. Gingival condition was scored on a scale from 0 to 3: 0 = healthy gingiva; 1 = mild inflammation characterized by discoloration and edema without bleeding on probing; 2 = moderate inflammation with redness, edema, and bleeding on probing; and 3 = severe inflammation marked by pronounced redness, edema, ulceration, and spontaneous bleeding. The GI was evaluated and scored at four sites per tooth: buccal and lingual surfaces, each at the mesial and distal aspects. The degree of gingival inflammation was recorded accordingly.^16^ Mean GI scores were then calculated across all examined sites for each participant.

The plaque index (PI) was used to assess dental plaque accumulation. It was scored on a scale from 0 to 3: 0 = no plaque at the gingival margin; 1 = a film-like plaque at the gingival margin, not visible to the naked eye; 2 = moderate plaque accumulation at the gingival margin and adjacent tooth surface, visible to the naked eye; and 3 = heavy plaque accumulation at the gingival margin and adjacent tooth surface. The PI was evaluated and scored at four sites of each tooth: buccal and lingual surfaces, as well as the mesial and distal parts. Oral hygiene status was recorded accordingly.^17^ Mean PI scores were calculated across all examined sites for each participant.

Probing pocket depth (PPD) was measured at six sites per fully erupted tooth: mesial, mid, and distal sites on both the buccal and lingual surfaces. PPD was determined by measuring the distance from the gingival margin to the base of the gingival sulcus. The mean PPD score across all examined sites was calculated for each participant.

### Saliva collection and biochemical analysis

Unstimulated whole saliva samples were collected from all participants between 9:00 and 11:00 a.m. to minimize diurnal variation. Children were instructed to refrain from eating, drinking, or chewing gum for at least one hour before collection. Participants were seated comfortably and asked to allow saliva to pool in their mouths and then expectorate into sterile polypropylene tubes over a five-minute period.^18^ The total volume collected was recorded, and the salivary flow rate (ml/min) was calculated accordingly.

Immediately after collection, saliva samples were centrifuged at 6,000 rpm for 10 minutes at 4°C (EBA 20, Hettich, Germany) to remove cellular debris. The clear supernatants were carefully transferred to labeled Eppendorf tubes and stored at -80°C until biochemical analysis.

Salivary cotinine and S100A8/A9 concentrations were measured using commercially available enzyme-linked immunosorbent assay (ELISA) kits, following the manufacturers’ instructions under standardized conditions. Cotinine levels were determined using the Human Cotinine ELISA Kit (Cat. No. YLA1901HU; YL Biont, Shanghai, China; assay range: 0.5–80 pg/ml), while S100A8/A9 levels were assessed using the Human S100 calcium-binding protein A8 (S100A8) ELISA Kit (Cat. No. YLA1830HU; assay range: 2–600 ng/ml) and the Human S100 calcium binding protein A9 (S100A9) ELISA Kit (Cat. No. YLA1831HU; assay range: 2–600 ng/ml), respectively. Absorbance was measured at 450 nm using a microplate reader (Thermo Fisher Scientific, Multiskan FC, USA).

### Statistical analysis

All statistical analyses were performed using IBM SPSS Statistics version 23.0 (SPSS Inc., Chicago, IL, USA). Data were initially assessed for normality using the Shapiro–Wilk test. Descriptive statistics are presented as mean and standard deviation (SD) or median (Q1–Q3) for continuous variables, and as frequencies and percentages for categorical variables.

Comparisons of continuous variables between groups were performed using the Mann–Whitney U test or the Kruskal–Wallis test. Categorical variables were compared using the chi-square test. Correlations between salivary cotinine and S100A8/A9 levels were assessed using Spearman’s rank correlation coefficient (ρ). A p-value <0.05 was considered statistically significant for all analyses.

## Results

A total of 150 children aged 6–12 years participated in the study. The mean age of the participants was 9.40±2.02 years. Of these, 52% (n=78) were girls and 48% (n=72) were boys. No statistically significant differences were observed between the ETS-exposed and non-exposed (control) groups regarding age (p = 0.435) or sex distribution (p=0.870) (Table 1).

**Table 1:** Demographic characteristic of study groups.

	ETS non-exposed group	ETS-exposed group	p-value
**Age mean ± sd**	9.27 ± 2.06	9.53 ± 1.98	0.435
**Sex n (%)**			
Girls	40 (53.3)	38 (50.7)	0.870
Boys	35 (46.7)	37 (49.3)	

Parental age, sex distribution, educational level, and household income did not differ significantly between the ETS-exposed and control groups (p>0.05).

Oral hygiene practices of the children, including toothbrushing frequency, use of toothbrush and toothpaste, and frequency of dental visits, were similar between the ETS-exposed and control groups. No statistically significant differences were observed between groups for any of these parameters (p>0.05) (Table 2). To further evaluate the potential confounding effect of oral hygiene on salivary inflammatory markers, an intragroup analysis was conducted within the ETS-exposed group. Salivary S100A8 and S100A9 levels were compared according to toothbrushing frequency using the Kruskal–Wallis test. No statistically significant differences were observed in salivary S100A8 or S100A9 levels among children with different toothbrushing habits within the ETS-exposed group (p>0.05).

**Table 2:** Distribution of oral hygiene practices among children exposed and not exposed to environmental tobacco smoke.

		ETS non-exposed n (%)	ETS-exposed n (%)	p-value
**Does your child have a regular tooth**	Yes	26 (34.7)	27 (36)	
**brushing habit?**	No	49 (65.3)	48 (64)	0.864
**Does your child have their own**	Yes	59 (78.6)	65 (86.6)	
**toothpaste?**	No	16 (21.4)	10 (13.4)	0.188
	Twice daily	8 (10.6)	9 (12)	
**How often does your child brush their teeth?**	Once daily	17 (22.7)	18 (24)	0.822
	Occasionally	50 (66.7)	48 (64)	
	Every 6 months	7 (9.4)	6 (8)	
**How often do you take your child to the dentist?**	Once a year	11 (14. 6)	7 (9.4)	0.429
	When there is a complaint	57 (76)	62 (82. 6)	

'Statistically significant, Chi-Square test

Clinical parameters, salivary flow rate, and levels of cotinine, S100A8, and S100A9 in the ETS-exposed and non-exposed groups are presented in Table 3. No statistically significant differences were observed in PI, GI, PPD, or salivary flow rate between ETS-exposed and non-exposed children (p>0.05). Salivary cotinine levels were significantly higher in the ETS-exposed group than in the non-exposed group. Similarly, S100A8 and S100A9 levels were significantly elevated in children exposed to ETS compared to those not exposed (p<0.05).

**Table 3:** Clinical parameters, salivary flow rate, cotinine, and S100A8/A9 levels among children exposed and not exposed to environmental tobacco smoke.

	ETS non-exposed Median(Q1-Q3)	ETS exposed Median(Q1-Q3)	p-value
**PI**	1.36 (1.01-1.90)	1.36 (1.11-1.90)	0.431
**GI**	1.45 (0.89-1.90)	1.30 (0.98-1.92)	0.694
**PPD (mm)**	2.11 (1.72-2.42)	2.15 (2.01-2.48)	0.180
**Salivary flow rate (ml/min)**	0.45 (0.40-0.55)	0.45 (0.40-0.52)	0.269
**Cotinine (pg/ml)**	11.27 (9.06-13.24)	17.45 (15.01-21.79)	<0.001*
**S100 A8 (ng/ml)**	89.69 (72.36-103.06)	134.29 (116.29-163.26)	<0.001*
**S100 A9 (ng/ml)**	89.05 (72.71-111.08)	103.32 (77.55-120.64)	0.036*

GI: Gingival index, PI: Plaque index, PPD: Probing Pocket Depth •Statistically significant, Mann Whitney U test

Correlations between exposure intensity and salivary cotinine, S100A8, and S100A9 levels are shown in Table 4. A moderate, statistically significant positive correlation was observed between cotinine and S100A8 levels. The correlation between cotinine and S100A9 was weak and not statistically significant. Additionally, S100A8 and S100A9 levels showed a positive correlation (ρ=0.257, p=0.002). A positive and statistically significant relationship was also observed between exposure intensity and cotinine levels. Similarly, S100A8 levels showed a positive and significant correlation with exposure intensity. In contrast, S100A9 levels did not demonstrate a significant association with exposure intensity.

**Table 4:** Correlations between exposure intensity, salivary cotinine, S100A8, and S100A9 levels.

		Cotinine	S100A8	S100A9
**Cotinine**	**ρ *p-value***		0.560** **< 0.001**	0.129 0.117
**Exposure intensity**	***ρ p-value***	0.214* **0.029**	0.213* **0.030**	0.031 0.751

"Correlation is significant at the 0.01 level 'Correlation is significant at the 0.05 level

## Discussion

Children exposed to ETS exhibit increased biological vulnerability, as its toxic components induce oxidative stress and activate early systemic and local inflammatory pathways. This process increases susceptibility to oral inflammatory changes even before clinical symptoms appear.^19^ Since saliva is a noninvasive and child-friendly diagnostic medium, salivary biomarkers offer an ideal tool for detecting these subtle biological effects.^20^ Cotinine, a stable metabolite of nicotine, provides a reliable and noninvasive marker of ETS exposure in saliva,^21^ while S100A8/A9 proteins act as key mediators and sensitive biomarkers of early inflammatory responses.^22^ Therefore, evaluating the impact of ETS exposure on salivary cotinine and S100A8/A9 levels in children is crucial for identifying early biochemical alterations. To our knowledge, no previous study has investigated this relationship in pediatric populations. This study thus aimed to determine whether ETS exposure is associated with changes in these biomarkers, reflecting early oral inflammatory responses.

This study found no significant differences between the ETS-exposed and non-exposed groups regarding age, sex distribution, parental educational level, or household income, indicating that these groups were well balanced demographically. Because socioeconomic and household characteristics are established predictors of children’s ETS and cotinine levels, demonstrating this balance reduces the risk of confounding.^23,24^ Similarly, oral hygiene habits, including toothbrushing frequency, use of personal toothpaste, and frequency of dental visits, were comparable between groups, minimizing behavioral confounding for oral biomarker comparisons.^4^ Moreover, intragroup analysis within the ETS-exposed group revealed no significant differences in salivary S100A8 and S100A9 levels based on toothbrushing frequency, suggesting that variations in oral hygiene practices alone were unlikely to account for the observed elevations in these biomarkers. Taken together, these findings reinforce the interpretation that differences in salivary inflammatory markers observed in this study primarily reflect the biological impact of ETS exposure rather than demographic, socioeconomic, or oral hygiene-related factors.

No statistically significant differences were observed in PI, GI, and PPD between children exposed to ETS and those not exposed. This finding is consistent with the meta-analysis by Oliveira, et al.^25^ (2022), which reported that evidence linking secondhand smoke exposure to clinically measurable periodontal deterioration in children and adolescents remains inconclusive, largely due to heterogeneity in exposure levels, age ranges, and assessment methods. The absence of significant clinical differences in our study may therefore reflect the relatively young age of the participants and the limited cumulative duration of ETS exposure—factors that can delay the manifestation of detectable periodontal changes. Importantly, previous research has emphasized that tobacco-related oxidative stress and inflammatory activation may occur well before overt clinical signs emerge, suggesting that early biochemical responses often precede measurable alterations in periodontal indices.^26,27^ Thus, our results support the notion that salivary biomarkers may offer greater sensitivity than clinical measures for identifying the subclinical inflammatory impact of ETS in pediatric populations.

A key finding of this study was the significantly higher salivary cotinine levels observed in children exposed to ETS. Cotinine, the primary metabolite of nicotine, has a longer half-life than nicotine and is widely recognized as a sensitive and specific biomarker of recent tobacco exposure.^21^ The elevated salivary cotinine concentrations in the ETS-exposed group validate the accuracy of the questionnaire-based exposure assessment, consistent with previous pediatric studies demonstrating concordance between reported household smoking and measured salivary cotinine levels.^28, 29^

In addition to cotinine, S100A8 and S100A9 levels were significantly elevated in children exposed to ETS. From a biological perspective, these proteins, members of the S100 family, are key regulators of innate immune activation and oxidative stress, and their increased expression has been linked to early inflammatory responses in mucosal tissues.^30^ Previous studies have demonstrated that salivary S100A8/A9 levels are elevated in inflammatory conditions such as periodontitis, and that exposure to environmental pollutants and ETS increases markers of inflammation and oxidative stress.^12,26,27^ These findings support the hypothesis that ETS may induce subclinical inflammatory responses in the oral cavity. In this study, the moderate positive correlation observed between cotinine and S100A8 further supports this relationship, suggesting that as nicotine exposure increases, inflammatory activation becomes more pronounced. Although S100A9 also showed a positive association with S100A8, its weaker and non-significant correlation with cotinine suggests that S100A9 may be less sensitive to ETS exposure. This difference may reflect the distinct regulatory roles of these proteins in inflammatory responses, with S100A8 often exhibiting stronger upregulation under stress or inflammatory conditions. The relatively weaker response of S100A9 in ETS-exposed children may therefore be due to its context-dependent expression and modulation by additional biological factors, although direct evidence in pediatric oral inflammation remains limited. Additionally, the salivary flow rate did not differ significantly between groups, indicating that ETS exposure does not appear to affect salivary gland function in this population. This finding suggests that the observed increases in inflammatory biomarkers are unlikely to be attributable to changes in salivary flow.

Analysis of exposure intensity revealed a significant positive correlation between higher ETS exposure levels and both salivary cotinine and S100A8, supporting a potential relationship between smoke exposure and early inflammatory activity. In contrast, S100A9 did not show a significant association with exposure intensity, consistent with weaker correlations previously observed. These findings suggest that S100A8 and S100A9 may exhibit distinct response patterns to ETS exposure.

### Limitations

This study has several limitations that should be considered when interpreting the findings. First, the case-control design design precludes the establishment of causal relationships between ETS exposure and changes in salivary inflammatory biomarkers. Longitudinal studies are needed to determine whether the observed associations reflect sustained biological effects or short-term fluctuations.

Second, environmental exposure outside the home was not directly measured; however, salivary cotinine served as an objective biomarker reflecting overall exposure from both household and non-household sources. Additionally, salivary cotinine reflects only recent ETS exposure, providing a short-term snapshot rather than capturing long-term or cumulative exposure patterns. This limitation hinders the ability to evaluate chronic exposure effects or to distinguish between children who are consistently exposed and those who are exposed intermittently. Future studies incorporating repeated measurements or additional long-term exposure indicators may enable a more comprehensive assessment.

Third, the study included only systemically healthy children, as systemic inflammatory conditions could influence S100 protein levels. While this approach reduces the likelihood of major confounding, it limits the generalizability of the findings to populations with systemic illnesses or more severe inflammatory disorders, who may exhibit different biomarker responses to ETS exposure.

Fourth, ETS exposure was assessed using parent-reported questionnaires, which may be subject to recall or social desirability bias. Parents might underreport or inaccurately report their child’s exposure, potentially leading to misclassification. However, despite these limitations, cotinine levels in the study were consistent with questionnaire-based exposure categories, with higher concentrations observed in the ETS-exposed group and low levels in the non-exposed group. Future studies incorporating repeated or fully objective exposure assessments may further reduce reporting bias and strengthen exposure classification.

Fifth, ELISA analyses were conducted as single measurements. However, all samples were analyzed under identical experimental conditions, using the same assay kits and lot numbers, following standardized protocols, and processed by the same operator in a single analytical run, thereby minimizing inter-assay variability. Nonetheless, future studies incorporating replicate measurements may further enhance analytical precision.

Future research with longitudinal designs, larger sample sizes, and more detailed assessments of exposure duration and intensity may help clarify the long-term impact of ETS on oral and systemic health. Additionally, incorporating complementary biomarkers of oxidative stress and immune modulation may provide a more comprehensive understanding of biological pathways by which ETS induces inflammatory responses.

## Conclusion

This study reveals that although ETS exposure may not yet lead to clinically detectable oral inflammatory changes in children, it is associated with measurable biochemical alterations indicative of early inflammatory activity. Elevated levels of cotinine and S100A8 in exposed children highlight the sensitivity of salivary biomarkers in identifying subclinical effects of passive smoking. These findings emphasize the importance of preventive efforts and educational strategies aimed at improving parental awareness and reducing children’s exposure to ETS, as early inflammatory alterations may increase vulnerability to future oral health issues.
